# MDR/XDR/PDR or DTR? Which definition best fits the resistance profile of *Pseudomonas aeruginosa*?

**DOI:** 10.1097/QCO.0000000000000966

**Published:** 2023-09-26

**Authors:** Federica Cosentino, Pierluigi Viale, Maddalena Giannella

**Affiliations:** a Department of Medical and Surgical Sciences, University of Bologna; b Infectious Diseases Unit, IRCCS Azienda Ospedaliero-Universitaria di Bologna, Bologna, Italy

**Keywords:** difficult-to-treat resistance, extensively drug-resistant, multidrug resistance, pan-drug resistance, prognostic meaning, *Pseudomonas aeruginosa*, therapeutic management

## Abstract

**Purpose of review:**

The aim of this narrative review is to compare the prognostic utility of the new definition of difficult-to-treat resistance (DTR) vs. established definitions in patients with *Pseudomonas aeruginosa* infection to understand the therapeutic implications of resistance classification and its impact on clinical outcome.

**Recent findings:**

Among Gram-negative bacteria (GNB), *P. aeruginosa* (PA) is associated with high rates of morbidity and mortality, mostly related to its intrinsic capacity of developing antibiotic resistance. Several classifications of antibiotic resistance have been proposed in the last 15 years. The most common used is that from Magiorakos *et al.* including multidrug resistance (MDR), extensively drug-resistant (XDR) and pan drug resistance (PDR) according to the number of antibiotic classes showing *in vitro* activity. A further classification based on the resistance to specific antibiotic classes (i.e. fluoroquinolones, cephalosporins, carbapenem resistance) was also proposed. However, both of them have been criticized because of limited usefulness in clinical practice and for poor correlation with patient outcome, mainly in infections due to PA. More recently the new definition of difficult-to-treat resistance (DTR) has been proposed referring to nonsusceptibility to all first-line agents showing high-efficacy and low-toxicity (i.e. carbapenems, β-lactam-β-lactamase inhibitor combinations, and fluoroquinolones). Studies including large cohorts of patients with GNB bloodstream infections have confirmed the prognostic value of DTR classification and its clinical usefulness mainly in infections due to PA. Indeed, in the recent documents from the Infectious Diseases Society of America (IDSA) on the management of antibiotic resistant GNB infections, the DTR classification was applied to PA.

**Summary:**

DTR definition seems to identify better than MDR/XDR/PDR and single class resistant categories the cases of PA with limited treatment options. It requires periodic revision in order to remain up-to-date with the introduction of new antibiotics and the evolving pattern of resistance.

## INTRODUCTION

*Pseudomonas aeruginosa* (PA) has intrinsic resistance to many drug classes, the capacity to form biofilms and the ability to quickly acquire resistance upon exposure to antibiotics [1]. The latest report of the European network EARS-Net showed that, in 2020, 30.1% of isolates were resistant to at least one antibiotic among carbapenems, fluoroquinolones, ceftazidime, piperacillin-tazobactam, and aminoglycoside. The highest percentage of resistance was observed for fluoroquinolones (19.6%), followed by piperacillin/tazobactam (18.8%), carbapenems (17.8%), ceftazidime (15.5%) and aminoglycosides (9.4%) [2].

Multidrug resistance (MDR) definitions have changed over recent years. In a majority of studies, the classification adopted for antibiotic resistance was that proposed in 2008 by the US and European Centers for Disease Control and Prevention (CDC and ECDC) and published in 2012 by Magiorakos *et al.*[3]. Multidrug -resistance (MDR) was defined as nonsusceptibility to ≥1 agent in ≥3 antimicrobial categories; extensively drug-resistant (XDR) as susceptibility limited to ≤2 categories; pan drug resistance (PDR), as nonsusceptibility to all agents in all antimicrobial categories [3]. Despite their advantages for epidemiological studies, these definitions have some limitations; indeed, they weigh all antibiotics equally only considering their *in vitro* activity, regardless of their “real-life” effectiveness, pharmacokinetic/pharmacodynamic behaviors, and toxicity, limiting the bedside applicability of MDR and XDR categories. To fill this gap, in 2015 fluoroquinolone resistance (FQR), extended-spectrum cephalosporin resistance (ESCR), and carbapenem resistance (CR) definitions were introduced by CDC [4,5]. Generally, in Enterobacterales and/or *Acinetobacter baumannii* infections, CR depicts cases with very limited treatment options. However, this could not be the case for PA where activity of FQ, ESC and/or β-lactam/β-lactamase inhibitor (BL/BLI) could be maintained also in presence of CR.

More recently, a new definition of resistance for Gram-negative infections has been proposed and labeled as difficult-to-treat resistance (DTR). This is based on the concept that nonsusceptibility to all first-line agents very often leads to use of second-line agents (such as aminoglycosides, tigecycline, or polymyxins) which are characterized by poorer pharmacokinetic properties and increased risk of toxicity, resulting in a better prediction of poor outcome. Validation on large patient cohorts has shown that this new definition is promising in better defining the correlation with clinical outcomes, and potentially in designing and evaluating clinical trials on the therapeutic management of antibiotic resistant Gram-negative infections [6].

Therefore, the aim of this narrative review is to examine and update available evidence about the prognostic utility of the new definition of DTR compared with the established definitions, in particular in patients with PA bloodstream infections (BSIs). 

**Box 1:**
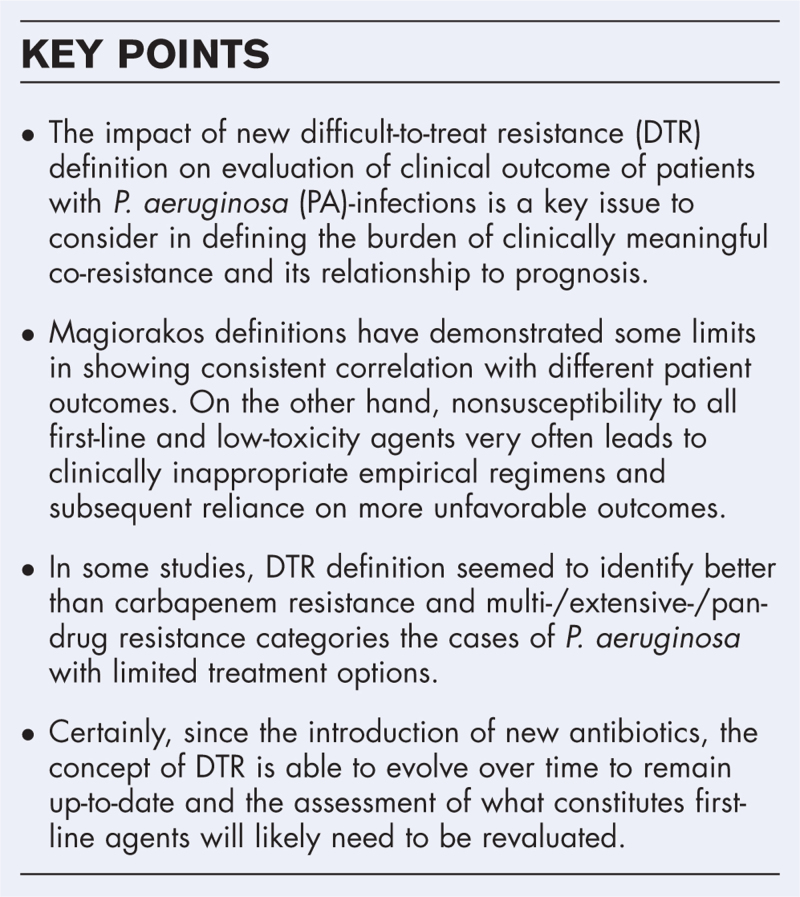
no caption available

## METHODS

We searched for randomized controlled trials (RCTs) and observational studies published from January 2022 to June 2023 on PUBMED using the following keywords

“difficult to treat resistance” or “difficult to treat definition”, “Gram-negative bloodstream infection”, “*P. aeruginosa* bloodstream infection”. Title and abstract screening were performed in order to check for consistency with the selected topic. Only studies published as full-text documents were reviewed.

### Impact of difficult-to-treat resistance vs. Magiorakos definition on mortality

The major issue about the classification of resistance from Magiorakos *et al.* is that its use does not well correlate with clinical implications [7^▪▪^,^8^]. After its introduction, only few studies have compared the prognostic utility of the new definition of DTR in Gram-negative BSI (GN-BSI) with the previous one, which main findings are summarized in Table 1.

**Table 1:** Summary of the main studies comparing the prognostic value of DTR category vs. MDR/XDR/PDR and/or CR categories in patients with Gram negative BSI

References	Country/period	No. of isolates	Etiological distribution of main pathogens	Prevalence of and mortality in MDR/XDR/PDR categories	Prevalence of and mortality in CR category	Prevalence of and mortality in DTR category	Multivariable analysis	Notes
Kadri *et al. CID* 2018	US, 2018	46 521	*E. coli* 28 640 (61.6%), *Klebsiella* spp. 9168 (19.7%), *Enterobacter* spp. 3221 (6.9%), *P. aeruginosa* 4493 (9.6%), *A. baumannii* 999 (2.14%)	NA CDC-defined resistance phenotypes: -prevalence: ECR 4165 (9.0%), FQR 10 240 (22%) -mortality: ECR 609 (15%), FQR 795 (7.7%).	Prevalence: 1048 (2.3%) Mortality: 183 (17.4%)	Prevalence: 471 (1.1%) Mortality: 202 (43%)	After adjustment for confounders, all resistant phenotypes remained individually associated with an increased mortality risk: FQR (aRR, 1.17; *P* < 0.0001); ESCR (aRR, 1.13; *P* = 0.0006); CR (aRR, 1.16; *P* = 0.02); DTR (aRR, 1.4; *P* < 0.0001).	Adjusted risk did not differ significantly among CR, ECR, or FQR phenotypes (*P* ≥ 0.45 for all), whereas DTR had a 20% higher adjusted mortality risk relative to CR (aRR, 1.2; *P* = 0.02), ECR (1.2; *P* = 0.001), and FQR (1.2; *P* = 0.008).
Giannella *et al. OFID* 2019	Italy, 2019	1576	*E. coli* 941 (59.7%), *K. pneumoniae* 326 (20.7%), *Enterobacter* spp. 77 (4.9%), *Proteus* spp. 55 (3.5%), *P. aeruginosa* 130 (8.2%), *A. baumannii* 33 (2.1%), *S. maltophilia* 14 (0.9%)	Prevalence: MDR 345 (21.9%) XDR 139 (8.8%) PDR 7 (0.4%) Mortality: MDR 50 (14%) XDR 32 (23%) PDR 2 (28.5%)	Prevalence: 207 (13.1%). Mortality: 53 (25.6%)	Prevalence: 174 (11%). Mortality: 45 (26%)	At multivariable analysis, the independent risk factors for all cause 30-day mortality were Charlson index, SOFA score, septic shock, CVC-related BSI, BSI due to CRE or NF-GNB and complicated BSI.	After incorporation of the resistance definitions into the baseline mortality model, the discrimination of the multivariate model for predicting 30-day mortality improved: 9%, 10% and 11% for DTR, Magiorakos at., and CR definitions, respectively.
Huh *et al. CID* 2020	Korea, 2020	1167	*E. coli* 390 (33.4%), *K. pneumoniae* 292 (25%), *P. aeruginosa* 288 (24.7%), *A. baumannii* 197 (16.9%)	Prevalence: MDR 286 (24.5%). Mortality: 100 (35%) CDC-defined resistance phenotypes: -prevalence: ESCR 216 (18.5%), FQR 99 (8.5%) -mortality: ESCR 41 (18.5%), FQR 13 (13%)	Prevalence: 218 (18.7%) Mortality (available only for CR+/DTR-): 14/71 (19.7%)	Prevalence: 147 (12.6%) Mortality: 74 (50%)	In a multiple logistic regression model adjusted for potential confounders, DTR was independently associated with mortality (aOR, 3.58; *P* = 0.016). CR (aOR, 0.83; *P* = 0.712), ESCR (aOR, 1.20; *P* = 0.576), FQR (aOR, 0.98; *P* = 0.958) showed no significantly increased mortality risk.	The authors divided the resistance phenotypes into 5 hierarchical nonoverlapping categories: DTR; CR but not DTR (CR+/ DTR−); ESCR but not CR or DTR (ESCR+/DTR−); FQR but not ESCR or CR (FQR+/ESCR-CR−); and others.
Yuan *et al. Front Microbiol* 2023	China, 2023	274	*P. aeruginosa* 274 (100%)	Prevalence: MDR-PA 103 (38%) Mortality: MDR-PA 41 (40%) The proportion of MDR-PA isolates were 93 (68%) in CR-PA group and 10 (7.3%) in CS-PA group (*P* < 0.001).	Prevalence: 137 (50%) Mortality: 54 (39%)	Prevalence: 46 (17%) Mortality: 23 (50%) The proportion of DTR-PA isolates were 46 (34%) in CR-PA group and 0 in CS-PA group (*P* < 0.001).	In multivariate cox regression, the 30-day crude mortality of CR-PA BSI was independently associated with MOF (HR 7.098; *P* < 0.001) and higher PBS (HR 1.092; *P* = 0.030), whereas receipt appropriate therapy improved prognosis (HR 0.312; *P* < 0.001).	No comparison between DTR vs. CR in terms of prognostic value on mortality.
Tabah *et al. ICM* 2023	International study (52 countries), 2023	2600	*E. coli* 272 (15.8%), *Klebsiella* spp. 482 (27.9%), *Enterobacter* spp. 141 (8.2%), *P. aeruginosa* 247 (14.3%), *A. baumannii* 350 (20.3%)	Prevalence: PDR 23 (0.9%). Mortality: Not known.	Prevalence: 635 (24.4%). Mortality: Not known.	Prevalence: 350 (13.5%) Mortality: 185 (52.8%)	Factors statistically significant for death in the multivariable analysis were infrequent clinical pharmacist consultation (OR 1.69), older age (OR 2.5), severity of illness at HA-BSI (OR 2.26), DTR GNB (OR 1.48) and required but not achieved source control (2.51).	No comparison between DTR vs. CR in terms of prognostic value on mortality.

BSI, bloodstream infections; CR, carbapenem resistance; CRE, carbapenem-resistant Enterobacteriaceae; CS, carbapenem-sensitive; CVC, central venous catheter; DTR, difficult-to-treat resistance; ESCR, extended-spectrum cephalosporin resistance; FQR, fluoroquinolone resistance; GN-BSIs, Gram-negative bloodstream infections; HA, hospital acquired; MDR, multidrug resistant; NF-GNB, nonfermentative Gram-negative; PA, *Pseudomonas aeruginosa*; PDR, pan drug resistant; SOFA, sequential organ failure assessment; XDR, extensively drug-resistant.

Kadri *et al.*[7^▪▪^] retrospectively analyzed a cohort of 29 474 inpatients with GNB-BSIs at 173 US hospitals. A total of 46 521 isolates were recorded: 28 640 (61.6%) *Escherichia coli*, 9168 (19.7%) *Klebsiella* spp., 3221 (6.9%) *Enterobacter* spp., 4493 (9.6%) *P. aeruginosa*, 999 (2.14%) *A. baumannii*. Among the isolates, the DTR prevalence was 1.1% (*n* = 471), compared with 1048 (2.3%) CR; 4165 (9.0%) ESCR; and 10 240 (22%) FQR. *P. aeruginosa* had a CR/DTR prevalence ratio of 4.5, reflecting the underlying susceptibility of many CR isolates to piperacillin-tazobactam (85.1% susceptible) and/or aztreonam (49.5% susceptible). Prevalence differences between CR and DTR were smaller but still significant for the other Gram-negative bacteria. Unadjusted mortality rate was 43% (202 of 471) in patients with DTR, 35% for CR (183 of 526), 22% for ESCR (609 of 2756) and 18% (795 of 4342) for FQR. After adjustment for confounders, all resistant phenotypes remained individually associated with an increased mortality risk compared with nonresistant GN-BSIs: FQR [adjusted risk ratio (aRR), 1.17; 95% confidence interval (CI) 1.10–1.26; *P* < .0001]; ESCR (aRR, 1.13; 1.06–1.22; *P* = .0006); CR (aRR, 1.16; 95% CI 1.03–1.31; *P* = .02); DTR (aRR, 1.4; 95% CI, 1.2–1.6; *P* < .0001). Adjusted relative mortality risk did not differ significantly among CR, ESCR, or FQR phenotypes (*P* ≥ 45 for all), whereas DTR had a 20% higher adjusted mortality risk relative than CR (aRR, 1.2; 95% CI, 1.0–1.4; *P* = 0.02), ESCR (1.2; 1.1–1.4; *P* = .001), and FQR (1.2; 1.0–1.4; *P* = 0.008) [7^▪▪^].

In our cohort of 1576 patients with GN-BSI, we [9] found that the most common causative microorganisms were *E. coli* 941 (59.7%), *K. pneumoniae* 326 (20.7%), *Enterobacter* spp. 77 (4.9%), *Proteus* spp. 55 (3.5%), *P. aeruginosa* 130 (8.2%), *A. baumannii* 33 (2.1%), *S. maltophilia* 14 (0.9%). The prevalence of DTR was 11% (*n* = 174) and, whereas among *K. pneumoniae* and *A. baumannii* BSIs, CR and DTR rates were comparable, they differed in *P. aeruginosa*. Indeed, DTR seemed to identify better than CR and XDR categories the cases of *P. aeruginosa* with limited treatment options. At multivariate analysis, the independent risk factors for all-cause 30-day mortality were Charlson index, SOFA score, septic shock, CVC-related BSI, BSI due to carbapenem-resistant Enterobacterales (CRE) or nonfermenting Gram-negative bacilli (NF-GNB), and complicated BSI. All the Magiorakos (MDR/XDR/PDR), CR, and DTR definitions were associated to a reduction of 30-day survival. When the resistance definitions were incorporated into the baseline mortality model, they significantly improved discrimination of the multivariate model for predicting 30-day mortality; the net reclassification improvement was 9%, 10%, and 11% for DTR, MDR/XDR/PDR, and CR definitions, respectively. Probably, the CR category was shown to have the highest impact on predicting survival because of the high prevalence of CRE in our cohort and the low number of PA cases [9].

**FIGURE 1:**
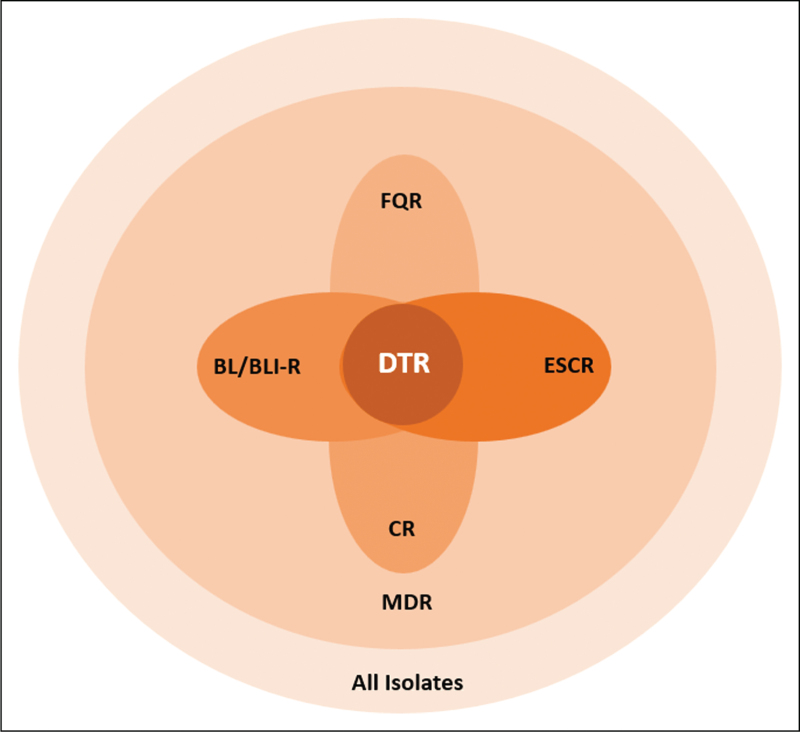
Schematic relationship of DTR with CDC-defined co-resistance phenotypes (adapted from Kadri SS *et al.* Difficult-to-treat resistance in Gram-negative bacteremia at 173 US hospitals: retrospective cohort analysis of prevalence, predictors, and outcome of resistance to all first-line agents. *Clin Infect Dis*. Published online July 23, 2018. doi:10.1093/cid/ciy378).

Similarly, Huh *et al.*[10] analyzed the impact of DTR on the 30-day mortality on a cohort of 1167 patients with GN-BSIs and compared DTR with traditional resistance classifications to determine their relative associations with clinical outcomes. The distribution of isolates was: *E. coli* 390 (33.4%), *K. pneumoniae* 292 (25%), *P. aeruginosa* 288 (24.7%), and *A. baumannii* 197 (16.9%). Overall, 147 (12.6%) were categorized as DTR, 17.7% of which were *P. aeruginosa* species. A multivariable model showed that only DTR, but not other categories, was significantly associated with mortality [adjusted odds ratio (aOR) 3.58, 95% CI 1.27–10.19]. DTR was also a significant predictor for mortality in the analysis of propensity score–matched cohorts (aOR 3.48, 95% CI 1.82–6.79). It is worth mentioning that in this study the authors divided the resistance phenotypes into five hierarchical nonoverlapping categories: DTR; CR but not DTR (CR+/DTR−); ESCR but not CR or DTR (ESCR+/DTR−); FQR but not ESCR or CR (FQR+/ESCR-CR−); and others. However, in real life resistance to multiple antibiotic classes can overlap (see Fig. 1).

More recently, Yuan *et al.*[11] conducted a 9-year multicenter retrospective study, enrolling 274 patients with PA-BSIs. Risk factors and prognosis of CR-PA BSI were identified, and CR-PA and DTR-PA rates were analyzed. Overall, 103 (38%), 137 (50%) and 46 (17%) strains were classified as MDR, CR and DTR PA. The proportion of MDR-PA and DTR-PA isolates were notably higher in CR-PA than in carbapenem susceptible (CS)-PA group: 68% vs. 7.3% (*P* < 0.001), and 34% vs. 0 (*P* < 0.001), respectively. All-cause 30-day mortality rates were 40%, 39% and 50% in MDR, CR and DTR-PA BSI, respectively. As expected, the 30-day survival probability of patients with CR-PA BSI was significantly worse than in patients with CS-PA BSI (63.6% vs. 77.3%, *P* = 0.014), such difference in survival was even higher between DTR-PA BSI vs. non-DTR-PA BSI patients (52.3% vs. 73.6%, *P* = 0.002).

Tabah *et al.*[12^▪▪^] carried out a prospective international study in a cohort of 2600 patients from 333 ICUs in 52 countries. They found that HA-BSI was more frequently caused by Gram-negative (1726/2927; 59%), with *Pseudomonas* spp. representing the 14.3% (247) of the isolates. Among the other GN pathogens, *E. coli* 272 (15.8%), *Klebsiella* spp. 482 (27.9%), *Enterobacter* spp. 141 (8.2%), *A. baumannii* 350 (20.3%) was the more represented. CR rate was 84.6% (296/350) in *Acinetobacter* spp., 37.8% (182/482) in *Klebsiella* spp., 33.2% (82/247) in *Pseudomonas* spp. and 7.4% (20/272) in *E. coli.* Overall, 13.5% (350/2600) of isolates were DTR. Higher mortality was found in early ICU-acquired HA-BSI, respiratory sources, DTR Gram-negative bacteria or fungal infections, and in patients who did not receive adequate antimicrobials or source control, when feasible. DTR was confirmed as a significant negative prognostic factor at multivariable analysis.

To conclude, CR is generally associated with resistance to other high-efficacy, low-toxicity agents and therefore indirectly overlaps with DTR (see Fig. 1). However, when analyzed in very large cohorts, or when compared with CR episodes not exhibiting DTR, and/or in studies assessing only PA or with high PA prevalence [7^▪▪^,^10^,^11^,^12^^▪▪^] the DTR class was associated with an increased relative risk of mortality compared with other resistance definitions. For these reasons, we deem the definition of DTR adopted by Infectious Diseases Society of America (IDSA) [6] guidance documents is more appropriate than that of CR used by European Society of Clinical Microbiology and Infectious Diseases (ESCMID) guidelines [13] to address the best therapeutic options for PA.

### Underlying mechanisms of resistance in difficult-to-treat resistance *P. aeruginosa* isolates and implications for therapeutic management

Few data are available about the types and prevalence of main resistant mechanisms underlying DTR and CR phenotypes in PA isolates, and which antibiotics remain active against these strains.

In a recent international prospective cohort study of 972 CR-PA strains from hospitalized patients, a carbapenemase gene was detected in 211/972 (22%) isolates [14^▪▪^]. Carbapenemase-producing CR-PA isolates exhibited higher degrees of meropenem resistance than noncarbapenemase-producing CR-PA isolates and were more frequently resistant to other antipseudomonal drugs. KPC-2 was the most common carbapenemase detected. Other common carbapenemase genes were bla_VIM-2_ (*n* = 52, 25%), bla_NDM-1_ (*n* = 14, 7%), bla_IMP-1_ (*n* = 13, 6%), and bla_GES-5_ (*n* = 12, 6%). Only one isolate had a class D carbapenemase gene (bla_OXA-23_). Overall, 670 (69%) of 972 isolates showed *oprD* mutations more frequently recognized in noncarbapenemase-producing CR-PA isolates than in carbapenemase-producing CR-PA isolates (72% vs. 59%; *P* = 0.0003); isolated harboring *oprD* mutation were more likely to have meropenem minimum inhibitory concentrations (MIC) values of more than 32 μg/ml than those without oprD mutations (89% vs. 68%; *P* = 0.0002). On the other hand, the carbapenemase-producing CR-PA isolates were less likely to be susceptible to cefepime (7% vs. 42%), ceftazidime (3% vs. 39%), piperacillin-tazobactam (5% vs. 36%), ciprofloxacin (6% vs. 35%), and amikacin (37% vs. 85%) than noncarbapenemase-producing CR-PA isolates (*P* < 0·0001). No data were available for susceptibility to the new molecules. All-cause 30-day mortality was higher in patients with carbapenemase-producing CR-PA infections compared with noncarbapenemase-producing CR-PA infections (22% vs. 12%). This mortality difference persisted even after adjusting for confounders.

In a nationwide Italian survey on 935 PA isolates obtained from patients with BSI and/or lower respiratory tract infections (LRTIs) in the period September 2013–November 2014, Giani *et al.*[15] investigated the antimicrobial susceptibility profiles to several old antibiotics and to ceftolozane/tazobactam (TOL), also depicting the molecular epidemiology of carbapenemase-producing isolates. TOL was the most active agent (90.9%) along with amikacin (88%) and colistin (84.7%). Meropenem was active against 65% of isolates. Overall, 85 strains (9.1%) were resistant to CTZ. Of these, 48 (5.1%) were carbapenemases producers. The most common carbapenemases were VIM- and IMP-types enzymes [15]. Four (8.3%) carbapenemase producing strains were positive for a bla_GES-5_ gene.

Hernandez-Garcia *et al.*[16] assessed the *in vitro* activity of several new drugs, in particular imipenem/relebactam, in 474 *P. aeruginosa* isolates from critically ill patients in Spain and Portugal. Susceptibility was 93.7%, 93.5%, 93.2% and 66% for imipenem/relebactam, ceftazidime/avibactam, ceftolozane/tazobactam and imipenem, respectively. Overall, up to 21.5% of the isolates were classified as MDR, 23.8% as XDR, and 18.6% as DTR. Twenty-five (40.3%) of the 62 sequenced isolates were carbapenemase producers; these were associated with high rates of resistance to new drugs. The most frequent carbapenemase genes were GES-13 (*n* = 13), VIM-2 (*n* = 3) and KPC-3 (*n* = 2) in Portugal; and VIM-20 (*n* = 3), VIM-1 (*n* = 2), VIM-2 (*n* = 1) and VIM-36 (*n* = 1) in Spain. The GES-13-CC235 clone was highly associated with XDR/DTR phenotype. Among noncarbapenemase-producing strains (59.7%; 37/62), the most frequent mutated genes were: Opr porin genes; QRDR genes; AmpC regulators; efflux pump-encoding genes and regulators and LPS modification genes.

Lasarte-Monterrubio *et al.*[17] evaluated the activity of cefiderocol, imipenem/relebactam, cefepime/taniborbactam and cefepime/zidebactam (these two last molecules are still not introduced in clinical practice) against a collection of 30 molecularly characterized ceftolozane/tazobactam and/or ceftazidime/avibactam-resistant *P. aeruginosa* isolates from patients previously exposed to cephalosporins. The authors showed that cefiderocol, cefepime/taniborbactam, cefepime/zidebactam and imipenem/relebactam were able to overcome β-lactamase-mediated ceftolozane/tazobactam and ceftazidime/avibactam resistance in some PA strains. Among the isolates producing PDC, OXA-2 and OXA-10 variants conferring resistance to ceftolozane/tazobactam and ceftazidime/avibactam, cefiderocol was the most active agent, followed by imipenem/relebactam that was highly active against all isolates, except two carrying a VIM-20 carbapenemase. Cefepime/zidebactam and cefepime/taniborbactam displayed activity against, 83.3% and 73.3% of the strains evaluated, respectively. Resistance was observed in some strains with alteration of PBP3 or upregulation of mexAB-oprM or mexXY efflux pumps.

These studies confirm the high rate of *in vitro* activity for each of the new drugs recommended from IDSA guidelines for the treatment of DTR-PA infections [6]. In addition, clinical studies have shown their good levels of effectiveness, safety and tolerability [18,19,20]. Thus, probably the DTR classification for PA strains susceptible to such drugs could not be appropriate so far. On the other hand, these studies demonstrate that new resistance mechanisms to the new molecules are already emerging. Hence to maintain its usefulness, the DTR definition should be continuously updated referring to a resistance profile that can evolve according to the availability of new drugs and/or the emergence of new resistance mechanisms [7^▪▪^].

## CONCLUSION

In some studies, DTR definition seemed to identify better than CR and MDR/PDR/XDR categories those cases of *P. aeruginosa* infection with limited treatment options and highest risk of mortality. Probably because multidrug resistance per se is not associated with higher mortality when effective and safe antibiotics are used for definitive therapy [9,10]. In addition, as emphasized in their manuscript by Kadri *et al.*[7^▪▪^], “DTR is not a fixed phenotype but rather a flexible framework”, therefore the concept of DTR needs to evolve over time to remain up-to-date and the assessment of what constitutes first-line agents will likely need to be revaluated [21].

## Acknowledgements

*All authors made a substantial contribution to the article.*

### Financial support and sponsorship

*None.*

### Conflicts of interest

*F.C. no conflicts of interest. PV received fees as speaker from Pfizer, MSD, Angelini, Menarini and Shionogi. M.G. received fees as speaker from Pfizer, MSD, Shionogi and Menarini.*

## REFERENCES AND RECOMMENDED READING

Papers of particular interest, published within the annual period of review, have been highlighted as:

▪ of special interest
▪▪ of outstanding interest
